# A Review of Phage Therapy against Bacterial Pathogens of Aquatic and Terrestrial Organisms

**DOI:** 10.3390/v9030050

**Published:** 2017-03-18

**Authors:** Janis Doss, Kayla Culbertson, Delilah Hahn, Joanna Camacho, Nazir Barekzi

**Affiliations:** Old Dominion University, Department of Biological Sciences, 5115 Hampton Blvd, Norfolk, VA 23529, USA; jdoss003@odu.edu (J.D.); kculb004@odu.edu (K.C.); dhahn001@odu.edu (D.H.); jcama008@odu.edu (J.C.)

**Keywords:** bacteriophage, *Vibrio* phage, phage therapy, aquaculture

## Abstract

Since the discovery of bacteriophage in the early 1900s, there have been numerous attempts to exploit their innate ability to kill bacteria. The purpose of this report is to review current findings and new developments in phage therapy with an emphasis on bacterial diseases of marine organisms, humans, and plants. The body of evidence includes data from studies investigating bacteriophage in marine and land environments as modern antimicrobial agents against harmful bacteria. The goal of this paper is to present an overview of the topic of phage therapy, the use of phage-derived protein therapy, and the hosts that bacteriophage are currently being used against, with an emphasis on the uses of bacteriophage against marine, human, animal and plant pathogens.

## 1. Introduction

Bacteriophage are commonly referred to as phage and are defined as viruses that infect bacteria. Phage are ubiquitous and require a bacterial host. They are also the most abundant organisms found in the biosphere. Bacteriophage-bacterial host interactions have been exploited by scientists as tools to understand basic molecular biology, genetic recombination events, horizontal gene transfer, and how bacterial evolution has been driven by phage [1]. Recently, there has been a renewed interest in phage therapy where phage are used as novel therapeutic agents in treating pathogenic bacteria [2].

Phage were discovered independently by two different scientists: Frederick Twort, a British pathologist, in 1915 and again by Félix d’Hérelle, a Canadian microbiologist, in 1917 [3]. Phage therapy was first attempted by d’Herelle to therapeutically treat humans. Interestingly, these early trials of phage therapy initially yielded impressive results. However, the general findings were extremely controversial because the studies were plagued with problems such as inadequate quality controls and failure to include control groups for comparison [4]. Inconsistent results due to a lack of reproducibility resulted in a decreased interest in phage therapy [5]. In addition, the discovery and ease of use of many chemical antibiotics further decreased interest in phage therapy research in the United States. In the meantime, phage therapy investigations continued in the Soviet Union, Eastern Europe, and France.

In the early 1980s, a renewed interest in phage therapy took shape predominantly due to the increase in multi-drug resistant (MDR) pathogens and the desire to find alternative treatment methods to the use of chemical antibiotics [6]. As a result, new investigations were focused on the use of phage therapy in the treatment of human infections as well as in agriculture, veterinary science, industry, and food safety.

In this review, we will discuss the utility of phage therapy in a variety of investigations. We start the review with the lifecycle of bacteriophage, followed by methods by which bacteria have evolved mechanisms against phage infection. Then, we discuss the advantages and disadvantages of phage therapy, with a section on the general uses of phage to kill pathogens in plants, humans, and marine animals.

## 2. Bacteriophage Life Cycle

Recent publications have provided interesting evidence that challenge the notion that viruses are non-living (reviewed in [7]). In a recent publication by Erez et al., communication between viruses has been identified. These reported findings indicate a unique small-molecule communication system that controls lysis–lysogeny decisions in a temperate phage [8]. Another study reported the assembly of a nucleus-like structure during the viral replication of phage 201Φ2-1 in *Pseudomonas chlororaphis* suggesting that phage have evolved a specialized structure to compartmentalize viral replication [9]. These findings indicate that viruses may be parasitic organisms similar to bacteria and fungi that rely on hosts to complete their life cycles.

**Figure 1:**
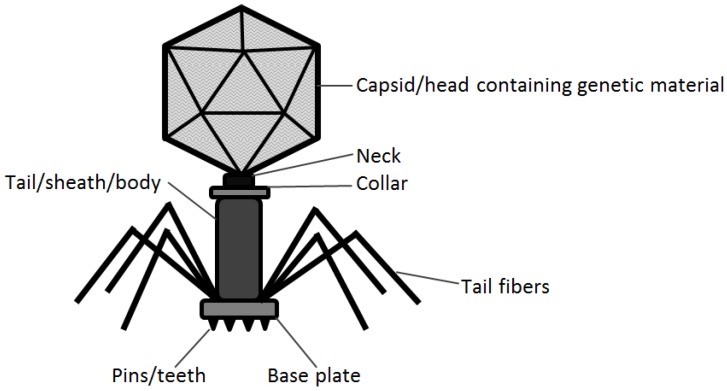
The anatomy of a tailed bacteriophage of the order *Caudovirales*.

These microscopic phage have beautifully diverse and complicated structures when observed through transmission electron microscopy. Two main features of tailed phage (order *Caudovirales*) include a capsid that encloses genetic material in the form of either DNA or RNA and a tail that varies in size among different bacteriophage (Figure 1).

**Figure 2:**
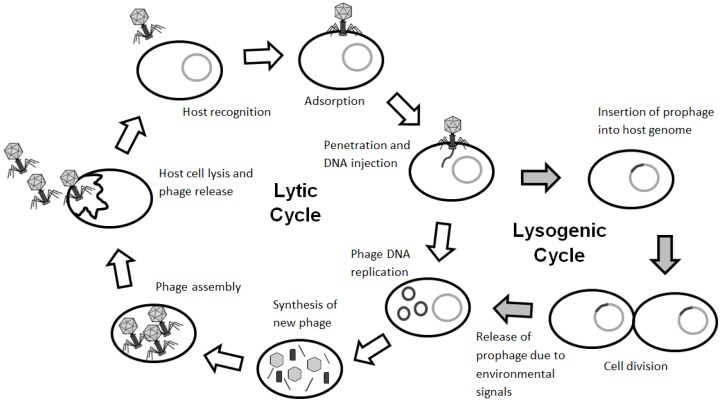
The phage life cycle. Lytic phage go through the lytic cycle, in which the host is lysed and progeny phage are released into the environment. Temperate phage can go through the lytic or the lysogenic cycle. Some phage rely on small molecules to communicate and execute lysis–lysogeny decisions [8]. In the lysogenic cycle, the phage genome is incorporated into the host genome; this phage DNA—now called a prophage—can be induced, leading to the expression of phage DNA and the lytic cycle.

Phage can undergo two different life cycles: the lytic cycle and the lysogenic cycle (Figure 2). Phage attach to the bacterial host specifically on a receptor found on the bacteria’s surface and injects its genetic material into the cell. The host cell provides the molecular building blocks and enzymes required to replicate the phage genetic material and produce progeny phage. Phage-encoded proteins such as endolysin and holin lyse the host cell from within. Holins are small proteins that accumulate in the cytoplasmic membrane of the host and allow endolysin to degrade peptidoglycan, allowing the progeny phage to escape. Subsequently, in the external environment, lytic phage can infect and destroy all neighboring bacteria. The production of large numbers of progeny by lytic phage is an advantage when lytic phage are used in phage therapy. However, lytic phage have narrow host ranges and infect specific bacterial species. This lack of a broad host range can potentially be overcome by using a phage cocktail. In the lysogenic cycle, temperate phage do not immediately lyse the host cell; instead, their genome is inserted into the host chromosome at specific sites. This phage DNA in the host genome is called a prophage, while the host cell containing the prophage is called a lysogen. The prophage is replicated along with the bacterial host genome, establishing a stable relationship. The disadvantage of using temperate phage in phage therapy is that some of the phage population insert their genome into the host chromosome and can lay dormant or alter the phenotype of the host. The lysogenic cycle can continue indefinitely unless the bacteria are exposed to stress or adverse environmental conditions. The induction signals vary among bacteriophage but prophage are commonly induced when bacterial SOS responses are activated due to antibiotic treatment, oxidative stress, or DNA damage [10]. Once the lysogenic cycle is terminated, expression of phage DNA ensues and the lytic cycle starts. Recently, phage that infect *Bacillus* species have been found to rely on small molecules termed “arbitrium” to communicate and execute lysis–lysogeny decisions [8]. The biological implication of this communication system is significant and explains that when a single phage encounters a large number or colony of bacteria, there are plenty of hosts to infect, favoring activation of the lytic cycle. As the host numbers become limited, it is more beneficial for the progeny phage to become dormant and enter lysogeny. These recent findings are worth pursuing further to determine if similar communication peptides are used by other bacteriophage or if cross-talk is evident among different bacteriophage.

## 3. Bacteria Have Evolved Mechanisms to Counter Phage Infection

Bacteriophage pose a constant threat to bacteria because of their overwhelming abundance and their ability to adapt to better infect their bacterial host. As a result, bacteria have evolved certain antiviral defenses that allow them to respond to phage infection. These defenses include modifications to surface receptors, super infection exclusion systems, restriction modification systems, and abortive infection [11]. Approximately one-half of all bacterial strains and the majority of Archaea have been discovered to have an interesting immunity system termed clustered regularly interspaced short palindromic repeats (CRISPR) and CRISPR-associated sequences (*cas*) that allow the bacteria to be resistant to and counteract some mobile genetic elements, including phage and plasmids [12].

Interestingly, phage have evolved to contain a similar CRISPR-Cas system, which was found in a cluster of bacteriophage O1-specific *Vibrio cholerae* phage, called the ICP1-related phage. These phage were isolated for the International Centre for Diarrhoeal Disease Research in Bangladesh (ICDDR, B). Of the eleven isolated phage, five were found to contain a CRISPR-Cas system [13]. The origin of the genes is unknown because the current pandemic *V. cholerae* serogroup O1, El Tor, does not contain a CRISPR-Cas system, though an L1 classical biotype strain *V. cholerae* O395 does have a specific type I CRISPR-Cas system [13]. The process of coevolution is significant and impacts bacterial and phage diversity. This is an important topic in the context of phage therapy, since the process is dynamic and new phage need to be identified and isolated to keep pace with bacterial evolution.

## 4. Advantages of Phage Therapy

Phage therapy has a number of advantages over traditional antibiotic therapy. The isolation of phage is fast, relatively simple, and inexpensive [14]. Resistance to phage develops about ten times slower than antibiotic resistance [14]. Phage remain infective under very harsh environmental conditions and tend to continue replicating until the population density of the host bacteria has been significantly reduced [15]. These qualities indicate that phage therapy—as opposed to traditional chemical antibiotics—may require fewer or limited administrations while performing as well or better than conventional treatments. Most phage isolated to date have a relatively high level of specificity for their host. This advantage of phage reduces the risk of harming the natural microbiota of the human body and eliminating the side-effects associated with chemical antibiotics [14]. Phage therapy is also suitable for use in humans, since phage do not infect eukaryotic cells [14]. In addition, the safety of phage therapy has been demonstrated with minor or minimal side effects [4].

## 5. Challenges Facing Phage Therapy

In general, the use of phage therapy can be an effective treatment against bacterial infections. However, there are some important factors that present a challenge to the use of phage therapy as a mainstream antimicrobial. One drawback is the use of phage therapy against intracellular pathogens such as *Salmonella* species [6]. Intracellular pathogens have the advantage of surviving inside host cells, where they would presumably be inaccessible to phage due to the inability of phage to enter eukaryotic cells [6]. Currently, few studies have investigated this problem, although one study did find phage therapy to be effective against salmonellosis, indicating the potential use of phage therapy against intracellular pathogens [6].

Although phage are not direct pathogens of eukaryotic cells, the human immune system may recognize phage as foreign antigens and respond by producing phage-neutralizing antibodies [6]. Furthermore, administering high titers of phage to a patient may induce an extreme reaction such as anaphylaxis, although this negative side-effect has not been observed [6].

Another characteristic of phage that may be a disadvantage in phage therapy is their ability to pick up genetic material through horizontal gene transfer. The use of phage therapy could lead to the transfer of genes that increase the bacterial host’s virulence through general or specialized transduction mechanisms [16]. Especially concerning is the possibility of transferring antibiotic-resistance genes and virulence factors; evidence suggests that genes for antibiotic resistance have been found in the genomes of some phage [17]. Infection with the CTX Φ prophage increases the virulence of *V. cholerae* [17]. Some phage have the ability to pick up genetic elements through horizontal gene transfer that allow the phage to produce bacterial toxins, such as enterotoxins and exfoliating toxins [18]. In order to avoid this problem, phage that are unable to package host DNA would be ideal in phage therapy [5]. One method to determine if a phage carries any of these genes is to perform polymerase chain reaction (PCR) before their use in phage therapy [18]. As an alternative, horizontal gene transfer can be exploited to directly transfer lethal genes or genes that can make bacteria susceptible to certain antibiotics. M13 is a non-lytic filamentous bacteriophage that is not frequently used in phage therapy. M13 has been genetically modified to have multiple insertion sites that can accommodate different genetic fragments. Interestingly, Moradpour et al. constructed a modified M13-derived phage with a lethal catabolite gene activator protein, and demonstrated that the number of *Escherichia coli* O157:H7 in contaminated cow milk and Luria–Bertani media could be reduced when treated with their designer phage. This customization of phage genomes can allow for extended versatility in the employment of designer phage against pathogenic bacteria [19]. 

Phage are natural components of all ecosystems. However, the high phage concentrations used in phage therapy are likely to be greater than the concentrations of any single type of phage found in nature [17]. If the phage are introduced into the environment at higher than normal concentrations, this might create an imbalance that would impact ecological communities [17]. Unlike antibiotics, phage do not simply degrade over time. They are stable over a wide range of temperatures and they can multiply indefinitely if host bacteria are consistently available. Removal of phage from the environment after their inadvertent release would pose a significant problem.

Although phage resistance has been shown to occur in laboratory studies, it has not been reported to be a substantial problem in clinical studies. However, it is still one of the major concerns about phage therapy, because this would limit the usefulness of phage therapy against MDR pathogens [20]. Phage resistance occurs through a variety of mechanisms; for example, through CRISPR-Cas systems or through a change in the surface receptors to which phage must attach [20]. Since phage and bacterial hosts coevolve, new phage can theoretically be re-isolated from the environment. This is an advantage of phage discovery in comparison to the extensive time required for the development or discovery of many chemical antibiotics. In this regard, phage resistance would not be as problematic as drug resistance [4].

Phage cocktails are mixtures of multiple phage that infect the same bacterial host, and can be used to reduce the probability of bacteria evolving resistance to one phage. In addition, phage cocktails tackle the issue of using phage that have a narrow host range. The mixture of different phage makes treatment more effective against a specific strain or targeting multiple bacterial strains. Phage cocktails are predicted to slow the development of phage resistance, but not necessarily eliminate it. Another enticing prospect is the use of combinatorial therapy, where phage therapy is used in conjunction with antibiotics. If a single type of therapy is ineffective, presumably the alternative treatment can make up for that inadequacy. Along the same lines, a seasonal schedule for phage therapy may be implemented, similar to the method of changing the influenza vaccine each year. In this way, phage therapy would keep pace with bacterial evolution.

Another issue that arises with phage therapy is the possible downstream effects of lysing bacteria. When Gram-negative bacteria are lysed, the cellular components such as endotoxin may be released. This is a major problem currently associated with the use of certain antibiotics [5]. If a large amount of endotoxin is released into the body, fever or septic shock can occur, which could lead to death [16]. One technique to circumvent this issue is to engineer phage that are lysis-deficient. There are unique phage proteins that play roles in the lysis of bacterial cells: endolysin, holing, and virion-associated peptidoglycan hydrolase (VAPH). Endolysins are enzymes produced by the bacteriophage that mediate the release of progeny phage at the end of the lytic cycle through the degradation of peptidoglycan. Holins are small proteins that accumulate in the cytoplasmic membrane of the host and allow endolysin to escape to degrade peptidoglycan [21]. In contrast, VAPH proteins degrade peptidoglycan to aid in host penetration when initially infecting bacteria and are not as strong as the endolysin enzymes in therapeutic uses [22]. An endolysin-deficient phage engineered to lyse the bacterial cell and prevent a harmful immunological response from the endotoxins would be ideal [22]. Endolysin gene disruption has been performed on phage such as T4, whose host is *E. coli*, and P954, whose host is *Staphylococcus aureus* [22]. Despite not releasing progeny, the endolysin-deficient phage retained their lethality by creating a hole in the inner membrane of the bacteria via the holin [22].

A possible solution to concerns regarding the use of phage therapy is the use of phage enzymes such as endolysin and VAPH instead of whole phage [17]. Using phage proteins instead of whole phage potentially avoids many of the problems of using a constantly reproducing particle, such as horizontal gene transfer and environmental containment issues [17]. In general, phage cocktails, combinatorial therapy, or phage protein products may provide promising alternatives to antibiotics [21].

## 6. Phage Therapy in Plants

New phage are constantly being discovered and utilized in many different applications, including the use of phage therapy in agriculture. The renewed interest in treating diseased crops with phage therapy has yielded promising results, including the treatment of some antibiotic-resistant infections involved in bacterial blight on soybeans [23]. Several phage have been approved by the United States Food and Drug Administration (FDA) for use on crops destined for human consumption. Use of phage therapy in plants is still in the early stages. Companies and organizations are focused on discovery, isolation, and marketing of bacteriophage products to control bacterial pathogens in environmental, food processing, and medical settings in the hope of reducing product loss and production costs [17]. In food processing, phage-based products have been used for the decontamination and elimination of pathogens from food sources to reduce food-borne illness caused by bacteria. Several different products have been created that target common foodborne pathogens such as *Listeria monocytogenes*, *Salmonella*, and *E. coli* O157:H7.

Several studies have investigated the efficacy of phage therapy in crops. Two phage (𝜙RSSKD1 and 𝜙RSSKD2) have been isolated that infect the banana wilt pathogen *Ralstonia solanacearum*. These phage were able to infect seven of the nine tested strains of the pathogen [24]. Three phage were isolated that infect several isolates of the soybean blight pathogen *Pseudomonas syringae* pv. *glycinea* [23]*.* Rombouts et al. tested a phage cocktail of five phage against 41 different strains of *Pseudomonas syringae* pv. *porri* on leek leaves. The phage cocktail was able to infect all of the bacterial strains. However, the therapy did not fully prevent or treat infection in the leeks [25]. The investigators of this study used a single application of the phage cocktail and hypothesized that a second application of the cocktail may produce better results [25]. These investigations are important in understanding the potential uses of phage therapy in the agricultural setting.

In addition to phage cocktails, proteins produced by and isolated from bacteriophage have been investigated as alternatives to using complete and intact phage to treat crop infections. Phage lysozyme is a protein that can fragment the bacterial cell wall, and has been isolated from phage 𝜙Xo411 [26]. Although the host of this phage is *Xanthomonas oryzae* pv. *oryzae*, the lysozyme (Lyse411) was tested against several other bacterial strains [26]. Lyse411 effectively reduced bacterial concentration in both *Xanthomonas* and *Stenotrophomonas*.

Endolysin is a protein that phage use to degrade the cell wall of their host bacterium, and has also been investigated as a treatment for plant infections. Wittmann et al. transformed endolysin genes from phage CMP1 into tomato seeds. When the resulting plants were infected with *Clavibacter michiganensis*, the transgenic plants containing the endolysins showed less severe signs of infection when compared to the infected wild-type tomato plants [27]. The study revealed that the infection was avoided and that the transgenic plants were able to grow with less wilting than untransformed plants. These findings indicate that phage endolysin protein is not toxic to plants and can be extremely beneficial in warding off infection.

## 7. Phage Therapy in Marine and Terrestrial Animals

Although human clinical trials for phage therapy do occur, most studies have been conducted on non-human animals. An MDR strain of *E. coli* from a diabetic foot ulcer was used to isolate a phage, TPR7, which was then tested in a mouse model of infection [28]. One group of mice was treated once with TPR7 and the second group of mice was treated multiple times with gentamycin. Both conditions resulted in clearance of the bacterial infection on day two. In the untreated group, all mice were still infected on day seven, indicating that the phage treatment was as effective as multiple antibiotic dose treatments. [28]. Another study utilized phage MR-10 as a co-treatment for methicillin-resistant *S. aureus* (MRSA) in diabetic mice [29]. The phage-treated mice showed improvement and decreased bacterial burden, redness, and swelling compared to the control group. This result was similar to the conventional treatment of mice with the antibiotic linezolid. The most effective results were evident when the mice were treated with a combination linezolid and MR-10 treatment [29]. The results of these two studies indicate that phage therapy can be as effective as antibiotic therapy and that phage therapy may be considered as a possible alternative or concurrent treatment, especially when treating infections caused by MDR bacteria.

In the food industry, meat may become contaminated by pathogenic bacteria. Tainted chicken, pork, and beef have led to food poisoning and food-related disease. For example, the transportation of pigs from the holding pens to the processing plant can result in the final product being contaminated by *Salmonella* species. Wall et al. investigated this phenomenon and utilized an anti-*Salmonella* phage cocktail to attempt to prevent bacterial infections. The use of phage cocktails in small pigs allowed the pigs to ward off *Salmonella enterica* serovar Typhimurium infections and reduced *Salmonella* colonization by a two- to three-log reduction in the tonsils, ileum, and cecum of the animals [30]. This initial analysis in animals indicates the promise of phage therapy, phage cocktails, and use of combination therapy as a means to reduce the transmission of food-borne illness due to pathogenic bacteria.

The fish and shellfish industry has also been investigated to determine if phage therapy can eliminate harmful bacteria. The demand for fish and shellfish has reached record highs, while regulations on wild catches have become more stringent. These two factors combined have resulted in increased aquaculture production. The impact of pathogenic bacteria on farm-raised marine and freshwater food sources can result in a huge financial burden if the products are killed, damaged, or infected [31]. Wang et al. investigated the use of phage therapy against *Vibrio harveyi* in abalone infections. It was reported that two *Siphoviridae* phage isolated from *V. harveyi* strains significantly improved the survival rate of abalone (*Haliotis laevigata*) and that phage therapy is an effective treatment against vibriosis [32]. Furthermore, Prasad et al. used water samples from locations such as fish farms to isolate nine lytic phage against *Flavobacterium columnare,* which causes cottonmouth disease in fish. One of these phage, FCP1, was used to treat a highly infectious strain of *F. columnare* that was injected into catfish. The phage therapy was prepared in three different ways: immersion in a bath containing a high titer of phage, intramuscular injection of phage, and orally administered phage through phage-impregnated food. It was reported that all of the fish tested survived and there was a significant decrease in the *F. columnare* numbers on the fish [33]. This study was interesting and creative in the method of phage therapy administration, and indicates the potential for phage treatment against fish contaminated with pathogenic bacteria. This study also showed that where there are pathogens, there is a high chance of finding a phage that can kill that pathogen in the same location or environment.

There have been some promising studies on the use of phage therapy against pathogens that cause bleaching and white-plague-like disease in corals. Phage BA3, which is specific to the coral pathogen *Thalassomonas loyana*, was used to treat diseased corals. In the study, the progression of the white-plague-like disease and its transmission to nearby healthy corals was inhibited [34]. The versatility of phage therapy makes it attractive in uses for human health, agriculture, and protection of fragile ecosystems. In the future, this alternative to antibiotics may play an important role in both aquatic and terrestrial environments.

## 8. Phage Therapy in Humans

Currently, the prevalence of MDR bacteria is increasing, while our portfolio of drugs is becoming ineffective. The evolution of antibiotic resistance in bacteria has become a major issue in the medical industry as a direct result of the overuse and misuse of antibiotics; bacteria such as MRSA [35], vancomycin-resistant *Enterococcus* (VRE) [36], and emergence of extensively drug-resistant (XDR) and totally drug-resistant (TDR) strains of *Mycobacterium tuberculosis* [37] continue to pose significant medical threats. This increased drug resistance to some of the most potent antibiotics used today makes treatment extremely difficult and costly against MDR strains of bacteria. According to the Centers for Disease Control and Prevention (CDC), two million people become infected with antibiotic resistant bacteria, and approximately 23,000 people die on a yearly basis in the USA from antibiotic resistant bacterial infections. Although this process can be slowed down by prescribing antibiotics only to treat confirmed bacterial infections, the overall trend has not diminished. As the number of antibiotic resistant bacteria continues to grow, alternative methods must be developed to kill these bacteria effectively.

While most studies involving phage have been done using animal models, there has been some research involving human subjects. Studies by Bruttin and Brüssow [38] and Sarker et al. [39] both found that ingestion of T4 phage did not produce any major health effects, with only a few minor side effects reported (nausea, stomach pain, and sore throat). Recently, a study called Phagoburn was conducted in an attempt to use phage therapy to treat *Pseudomonas aeruginosa* and *E. coli* infections in human burn victims. The goal of the Phagoburn project is to reduce bacterial burden and decrease sepsis in patients afflicted with severe burns and infection. Despite setbacks, the project has been funded by the European Union and has begun phase I/II clinical trials in France, Belgium, and Switzerland [40]. Although this project is a great advancement in the study of phage therapy, further in vitro and clinical trials are still needed in order to gain widespread acceptance to use phage therapy to treat humans with pathogenic or MDR bacterial infections.

## 9. Conclusions

Phage therapy is an exciting rediscovered field that will surely provide countless benefits to science, agriculture, veterinary science, and medicine, including offering a possible solution to counteract the increased prevalence of antibiotic-resistant pathogens. The potential of combining antibiotic therapy and phage therapy, the use of phage cocktails, or the use of phage protein products may be the best areas for successful phage treatment of infections. Considering the utility and diverse applications of phage therapy, this area of research is extremely necessary. This review brings together a variety of considerations with regard to phage therapy and its possible utility. Promising results along with cautious examination of the safety and efficacy of phage indicate that phage therapy against pathogenic bacteria may be a future remedy against pathogens that infect humans, terrestrial animals, and marine animals.
